# Supplementary material for: Comparative study of Plasmodium falciparum msp-1 and msp-2 Genetic Diversity in Isolates from Rural and Urban Areas in the South of Brazzaville, Republic of Congo
Source: Pathogens. 2023 May 22;12(5):742. doi: 10.3390/pathogens12050742 (PMC10221593; doi:10.3390/pathogens12050742)
Supplement: Supplementary file 1 [file pathogens-12-00742-s001.zip › Supplementary figure1.pdf]

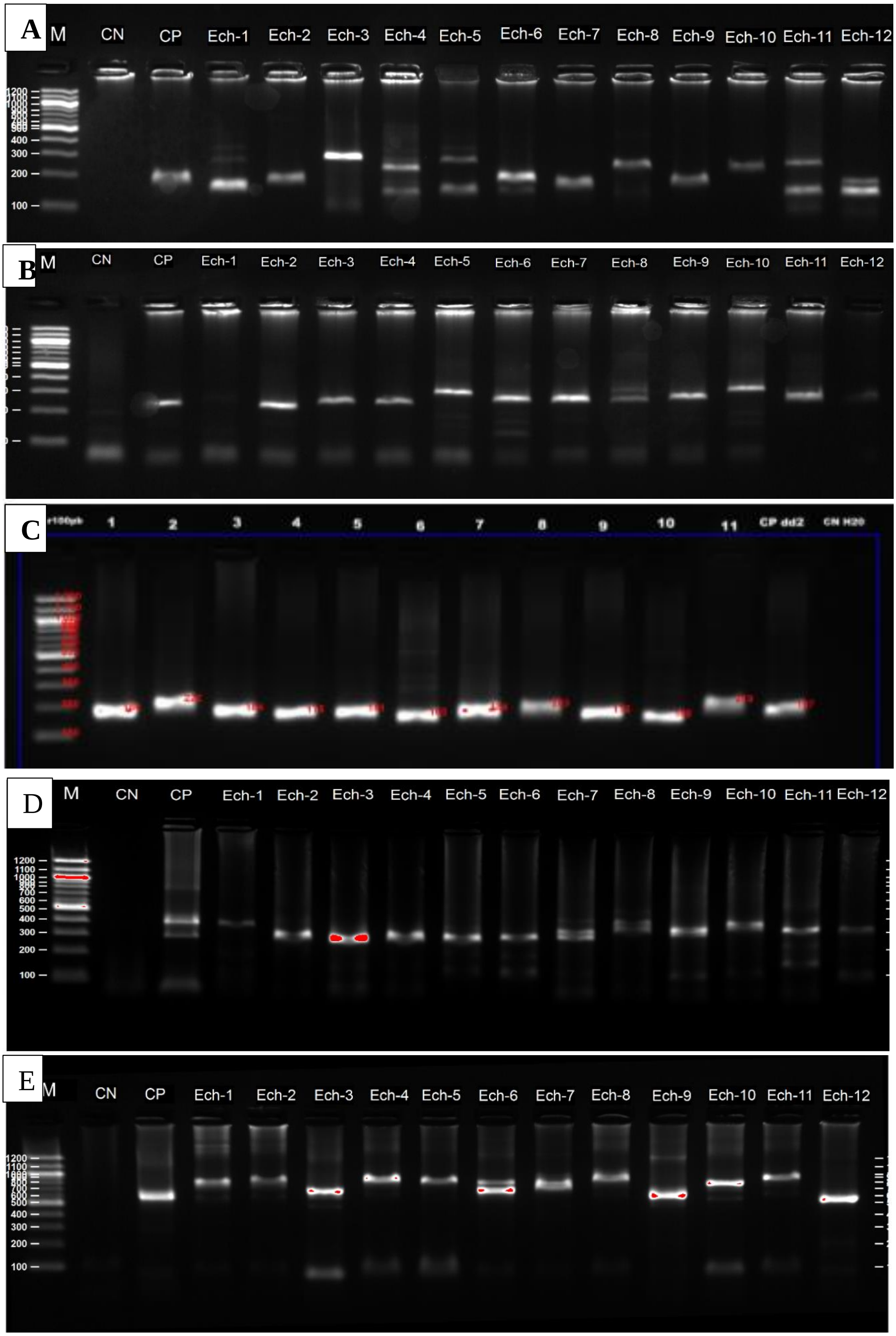

Figure S1: Electrophoreses of pfmsp1 and pf msp2 PCR showing the allelic families K1 in (**A**), MAD20 in (**B**), RO33 in (**C**), FC27 in (**D**) and 3D7 in (**E**)
