# Supplementary material for: Comparative study of Plasmodium falciparum msp-1 and msp-2 Genetic Diversity in Isolates from Rural and Urban Areas in the South of Brazzaville, Republic of Congo
Source: Pathogens. 2023 May 22;12(5):742. doi: 10.3390/pathogens12050742 (PMC10221593; doi:10.3390/pathogens12050742)
Supplement: Supplementary file 1 [file pathogens-12-00742-s001.zip › Supplementary tables.pdf]

Table S1: Primary and secondary PCR primers for *msp1 and msp2*

Gene	PCR round	Primer name	Sequence (5'-3')	An. T°	Positive control
Msp1	Primary	Lisa-1	ACATGAAAGTTATCAAGAACTTGTC	62°C	-
		Lisa-2	TACGTCTAATTCATTTGCACGAA		
	Secondary	K1-F	AAGAAATTACTAGAAAAGGTGCAAGTG	64°C	3D7
		K1-R	AGATGAAGTATTTGAACGAGGTAAAGTG		
		MAD-F	TGAATTATCTGAAGGATTTGTACGTCTTGA	67°C	HB3
		MAD-R	GAACAAGTGGAACAGCTGTTA		
		RO-F	GAGCAAATACTCAGGTTGTTGCAAAGC	45°C	Dd2
		RO-R	ACGATTTGCAGCACCTGGAGATCT		
Msp2	Primary	MSA2-1	ATGAAGGTAATTAAAACATTGTCTATTATA	62°C	-
		MSA2-4	ATATGGCAAAAGATAAAACAAGTGTTGCTG		
	Secondary	3D7-1	GCAGAAAGTAAGCCTTCTACTGGTGCT	55°C	3D7
		3D7-2	GATTTGTTTCGGCATTATTATGA		
		FC27-1	GCAAATGAAGGTTCTAATACTAATAG	55°C	Dd2
		FC27-2	GCTTTGGGTCCTTCTTCAGTTGATTC		

An. T°: Annealing temperature

Table S2: Allelic family's frequencies of msp1 according to socio-demographic characteristics in rural and urban area

Parameters	n1; n2	Rural				Urban			
		K-1	MAD-20	RO33	p-value	K-1	MAD-20	RO33	p-value
**Season**									
Dry	317; 126	101 (31.9)	85 (26.8)	131 (41.3)	0.0002	61 (48.4)	20 (15.9)	45 (35.7)	0.0839
Rainy	562; 136	243 (43.2)	160 (28.5)	159 (28.3)		58 (42.6)	37 (27.2)	41 (30.1)	
**Age group, (years)**									
< 5	82; 11	29 (35.4)	25 (30.5)	28 (34.1)	<0.0001	5 (45.5)	4 (36.4)	2 (18.2)	0.5568
[5 - 15[	278; 105	131 (47.1)	26 (9.4)	121 (43.5)		44 (41.9)	25 (23.8)	36 (34.3)	
> 15	441; 146	184 (41.7)	116 (26.3)	141 (32.0)		70 (47.9)	28 (19.2)	48 (32.9)	
**Type of infection**									
Microscopic	389; 67	146 (37.5)	120 (30.8)	123 (31.6)	0.2115	30 (44.8)	18 (26.9)	19 (28.4)	0.4026
Sub-microscopic	490; 194	198 (40.4)	125 (25.5)	167 (34.1)		89 (45.9)	38 (19.6)	67 (34.5)	
**Clinical status**									
Asymptomatic	233; 57	93 (40.0)	67 (28.8)	73 (31.2)	0.4234	19 (33.3)	19 (33.4)	19 (33.3)	0.5721
Symptomatic	156; 22	53 (34.0)	53 (34.0)	50 (32.0)		10 (45.5)	5 (22.7)	7 (31.8)	

n1: Total number of fragments for each parameter in rural area. **n2**: Total number of fragments for each parameter in urban area

Table S3: Allelic family's frequencies of msp2 according to socio-demographic characteristics in rural and urban area

Parameter	n1; n2	Rural			Urban		
		FC27	3D7	p-value	FC27	3D7	p-value
**Season**							
Dry	317; 51	194 (61.2)	123 (38.8)	0.1729	25 (49.0)	26 (51.0)	0.3900
Rainy	547; 161	360 (65.8)	187 (34.2)		90 (56.0)	71 (44.0)	
**Age group (years)**							
< 5	67; 9	32 (47.8)	35 (52.2)	0.0113	2 (22.2)	7 (77.8)	0.0955
[5 - 15[	353; 100	236 (67.0)	117 (33.0)		59 (59.0)	41 (41.0)	
> 15	444; 102	286 (64.4)	158 (35.6)		54 (53.0))	48 (47.0)	
**Type of infection**							
Microscopic	414; 92	259 (62.6)	155 (37.4)	0.3592	54 (58.7)	38 (41.3)	0.3119
Sub-microscopic	450; 118	295 (65.6)	155 (34.4)		61 (51.7)	57 (48.3)	
**Clinical status**							
Asymptomatic	254; 61	159 (62.6)	95 (37.4)	0.9839	34 (55.7)	27(44.3)	0.3184
Symptomatic	160; 30	100 (62.5)	60 (37.5)		20 (66.7)	10 (33.3)	

n1: Total number of fragments for each parameter in rural area. **n2**: Total number of fragments for each parameter in urban area
